# Strategic Facet Design of In_2_O_3_ Catalysts for Enhanced Kinetics and Hydrogen Suppression in Iron–Chromium Flow Batteries

**DOI:** 10.1002/advs.202512148

**Published:** 2025-10-08

**Authors:** Yinping Liu, Chao Guo, Fangang Qu, Yida Zhang, Kuo‐Wei Huang, Chunming Xu, Jia Guo, Quan Xu, Yingchun Niu

**Affiliations:** ^1^ State Key Laboratory of Heavy Oil Processing China University of Petroleum (Beijing) Beijing 102249 China; ^2^ College of Chemical Engineering Inner Mongolia University of Technology Hohhot 010051 China; ^3^ Chemistry Program Division of Physical Science and Engineering King Abdullah University of Science and Technology (KAUST) Thuwal 23955‐6900 Saudi Arabia; ^4^ Department of Chemistry Aarhus University Aarhus N 8200 Denmark

**Keywords:** crystal facet engineering, hydrogen evolution suppression, indium oxide (In_2_O_3_), in situ characterization, Iron‐chromium redox flow battery (ICRFB)

## Abstract

Iron‐chromium redox flow batteries (ICRFBs) show promise for large‐scale energy storage, but their performance is hindered by the hydrogen evolution reaction (HER) and sluggish anode Cr^3^⁺/Cr^2^⁺ redox kinetics. Here, an octahedral In_2_O_3_ catalyst with exposed high‐activity (222) crystal planes is reported, synthesized via high‐temperature solution thermal decomposition and grown in situ on carbon cloth. The catalyst is grown in situ on carbon cloth to form a nanostructured indium‐based electrode (In_2_O_3_‐TCC). Grazing incidence wide‐angle X‐ray scattering confirms In_2_O_3_ phase formation, while XANES reveals abundant oxygen vacancies (Ov) serving as anode reaction active sites. In_2_O_3_‐TCC exhibits enhanced electrochemical properties, including a tripled double‐layer capacitance (8.92 mF cm^−^^2^), a reduced charge transfer resistance (1.042 Ω), and improved Cr^3^⁺/Cr^2^⁺ kinetics. Density functional theory (DFT) shows that anode HER suppression arises from favorable H⁺ adsorption energy and a high desorption barrier. Furthermore, an in situ differential electrochemical mass spectrometer (DEMS) confirms effective anode HER suppression. The electrode achieves an energy efficiency of 84.02% at 140 mA cm^−^^2^ and stable performance over 500 cycles. This work offers a new pathway for designing high‐efficiency, long‐lifetime ICRFB electrodes.

## Graphical abstract

The electrode modified with octahedral indium oxide catalyst with exposed high activity (222) crystal plane can suppress the hydrogen evolution side reaction and improve the reaction kinetics of chromium of iron‐chromium flow batteries. The battery exhibits extremely high energy efficiency of 84.02% at an ultra‐high current density of 140 mA·cm^−2^.

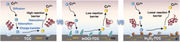

## 1 Introduction

As a transfer station between new energy generation and the power grid, large‐scale long‐term energy storage can solve the fundamental contradiction between the intermittency of renewable energy and the continuous and stable supply required by the power grid.^[^ ^1^ ^]^ In iron‐based flow batteries, iron vanadium redox flow batteries (RFBs)^[^ ^2^ ^]^ rely on scarce vanadium resources, all iron RFBs^[^ ^3^ ^]^ are plagued by cross‐contamination, and zinc iron RFBs^[^ ^4^ ^]^ face zinc dendrite problems. In contrast, iron chromium flow batteries (ICRFBs) have significant advantages, which using Fe^2^⁺/Fe^3^⁺ and Cr^3^⁺/Cr^2^⁺ as the redox pair. ICRFBs have significant advantages in meeting society's demand^[^ ^4^, ^5^ ^]^ for high‐quality, safe, reliable power sources and efficient electricity consumption due to their low raw material prices, long cycle life, easy scalability, and capacity decoupling.^[^ ^6^, ^7^, ^8^, ^9^ ^]^ However, the Cr^3^⁺/Cr^2^⁺ redox couple in the negative electrode of the ICRFBs shows poor electrochemical activities,^[^ ^10^ ^]^ and the potential is close to hydrogen evolution reaction (HER), which makes it prone to hydrogen evolution side reactions, resulting in low overall energy efficiency (EE) and poor cycle life of the battery.^[^ ^11^, ^12^ ^]^ Therefore, it is urgent to develop effective strategies to improve the reaction kinetics of Cr^3^⁺/Cr^2^⁺ and suppress HER, thereby enhancing the performance of ICRFBs.

Developing various types of catalysts to enhance reaction activity has always been a popular goal.^[^ ^13^ ^]^ At present, catalysts used for RFBs include metals such as bismuth,^[^ ^14^ ^]^ indium,^[^ ^15^ ^]^ lead,^[^ ^16^ ^]^ tin,^[^ ^17^ ^]^ metal oxides such as manganese oxide,^[^ ^18^ ^]^ cobalt oxide,^[^ ^19^ ^]^ and titanium dioxide,^[^ ^20^ ^]^ as well as carbon‐based catalysts such as carbon dots,^[^ ^21^ ^]^ graphene,^[^ ^22^ ^]^ carbon nanotubes,^[^ ^23^ ^]^ and biomass carbon.^[^ ^24^ ^]^ The catalytic performance of primitive carbon‐based catalysts is limited. Kim et al.^[^ ^25^ ^]^ heat‐treated carbon felt in an air atmosphere at 500 °C to introduce a large number of oxygen groups, improving the hydrophilicity and active sites of the electrode. He et al.^[^ ^26^ ^]^ chemically etched carbon nanotubes through KOH treatment, improving the graphitization degree and wettability of the sample. Liu et al.^[^ ^27^ ^]^ provided more active sites for the carbon nanotube structure by doping with B and N heteroatoms, and effectively activated the electrons of carbon atoms around the heteroatoms. To solve the problem of insufficient electron transfer performance of ICRFB electrodes, Niu et al. used SiO_2_ etching to construct a dense nanoporous structure on the surface of carbon cloth, and prepared a multifunctional carbon cloth electrode rich in vacancies,^[^ ^28^ ^]^ However, defect modified electrodes have no catalytic activity for specific redox reactions, especially for the slow kinetics of Cr redox reactions. The above improvement methods increase the active centers and hydrophilicity of carbon‐based catalysts,^[^ ^29^ ^]^ but their improvement on charge transfer processes and mass transfer kinetics is limited, and metal catalysts are still needed to provide active sites.

Compared to these carbon‐based materials, metals are high‐quality catalysts for redox reactions. Among various metals, indium is abundant in resources, non‐toxic, easy to process, and has excellent physical and chemical properties. So far, various indium‐based catalysts have been widely used in fields such as photocatalysis, water treatment,^[^ ^30^ ^]^ and electrochemical reduction of CO_2_,^[^ ^31^, ^32^ ^]^ et al. In terms of flow batteries, Wang et al.^[^ ^33^ ^]^ used In^3+^ modified graphite felt to demonstrate that the modified sample reduced the charge transfer resistance of the ICRFBs, but did not further investigate its catalytic mechanism. In our previous work,^[^ ^8^ ^]^ we directly added InCl_3_ catalyst to the electrolyte and found that its mode of action was to convert the charging process into In nanoparticles deposited on the surface of the carbon cloth electrode and dissolving into In^3+^ during the discharge process, thus cycling. However, indium metal itself has the problems of high cost and easy agglomeration.

**Figure 1:**
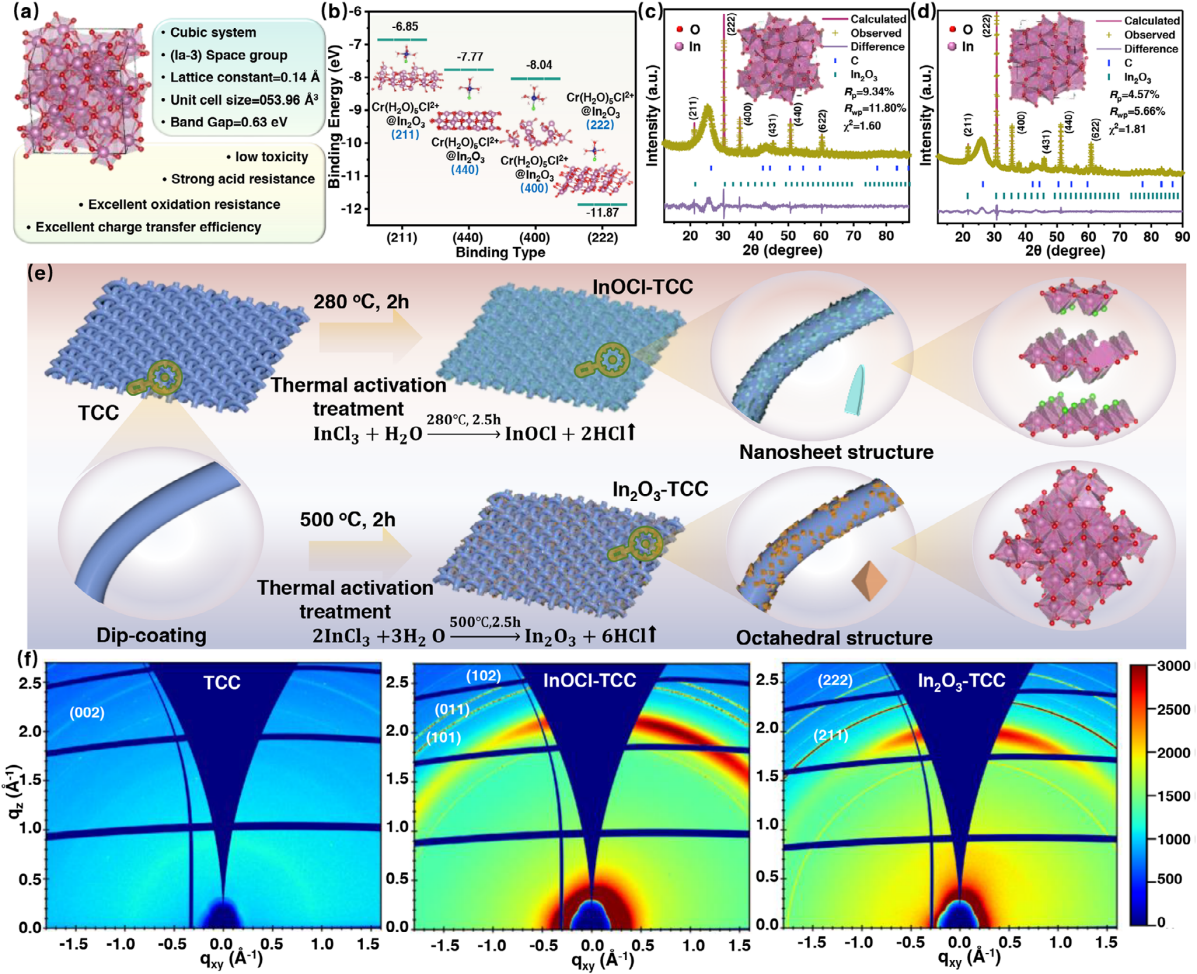
a) Crystallographic information and advantages of In_2_O_3_. b) The adsorption energy and adsorption configuration of Cr(H_2_O)_5_Cl^2+^ on the (211), (400), (440), and (222) surfaces of In_2_O_3_, respectively. Rietveld refinement of XRD patterns of c) octahedral In_2_O_3_ and d) spherical In_2_O_3_ prepared, with corresponding cell models shown in the illustrations. In these models, pink and red spheres represent In and O atoms, respectively. e) Schematic diagram of preparation of In‐based catalyst modified carbon cloth electrode. f) GIWAXS patterns of the TCC, InOCl‐TCC, and In_2_O_3_‐TCC electrodes.

Metal oxides have also become promising electrocatalysts because they are cheaper alternatives than precious metals.^[^ ^34^, ^35^ ^]^ Liang et al.^[^ ^36^ ^]^ prepared single‐crystal InOCl nanosheets and demonstrated their high photocatalytic activity in the degradation of organic dyes due to their open crystal structure and indirect light transitions. Yang et al.^[^ ^37^ ^]^ prepared InOCl with a sheet‐like structure at low temperature and obtained octahedral In_2_O_3_ at high temperature, which has the advantages of strong acid resistance, good oxidation resistance, low toxicity, and high charge transfer efficiency^[^ ^38^ ^]^ (**Figure** **1**a).

Furthermore, abundant oxygen vacancies (Ov) on the metal oxides surface, by providing active sites, optimizing electron transfer efficiency, and regulating the adsorption/desorption energy of reactants, have emerged as a critical factor in enhancing electrode catalytic performance, and has been explored in vanadium redox flow batteries (VRFBs). Huang et al.^[^ ^39^ ^]^ synthesized TiO_2_ nanosheets with tunable Ov concentrations, which optimized the desorption of V^3+^ and electron transfer processes, and clarified the strong correlation between Ov concentration and catalytic activity. Kabutamu et al.^[^ ^40^ ^]^ loaded NiWO_4_ nanowires rich in Ov onto the heat‐treated electrode of the VRFBs, which confirmed that abundant Ov and uniformly distributed nanowires are the key to performance improvement. In VRFBs, V⁵⁺ has strong oxidizing properties and is prone to undergo redox reactions with Ov on the electrode surface, capturing lattice oxygen from the Ov. Therefore, the research on Ov in VRFB mainly focuses on designing stable Ov to accelerate the reduction of V⁵⁺. In contrast, Ov in ICRFBs needs to lower the adsorption energy barrier of Cr^3^^+^ and increase the desorption potential barrier of H⁺, which poses a more precise requirement for the electron density distribution of Ov.

However, the performance of indium metal oxide with Ov in charge and discharge of ICRFBs has not been studied. And the adsorption capacity of Cr(H_2_O)_5_Cl^2+^ in the ICRFBs system varies greatly among different crystal faces of In_2_O_3_. The adsorption energy of Cr(H_2_O)_5_Cl^2+^ on the (211) crystal face is −6.85 eV, but the (222) crystal face can stably bind with Cr(H_2_O)_5_Cl^2+^, and its adsorption energy is greatly reduced to −11.87 eV (Figure 1b). The density of states (DFT) calculation (Figure S1, Supporting Information) shows that the overlap area between Cr‐3d and In‐5p orbitals in In_2_O_3_ (222) is much larger than that of (211), (400), and (440) crystal faces, which is beneficial for charge transfer in the anodic Cr^3+^/Cr^2+^ redox reaction. Therefore, accurately constructing In_2_O_3_ catalysts with exposed specific crystal planes on the electrode surface and studying the kinetics of the catalyst for the reaction is of great significance for improving battery performance.

Herein, we prepared an exposed high reactivity (222) crystal face In_2_O_3_ catalyst with a specific octahedral nanostructure by high‐temperature solution thermal decomposition method, and uniformly grown it in situ on the surface of a carbon cloth electrode (In_2_O_3_‐TCC) as a high‐performance catalyst for improving Cr^3+^/Cr^2+^ reaction activity and suppressing HER in the ICRFBs system (Figure 1e). Research through GIWAXS and XANES has shown that In_2_O_3_ with (222) crystal planes has been successfully loaded onto the surface of carbon cloth fibers, and its specific structure provides sufficient oxygen vacancies as specific active sites compared to sheet‐like InOCl catalysts and spherical In_2_O_3_ catalysts. Experimental and theoretical studies have shown that the C_dl_ of In_2_O_3_‐TCC is ≈3 times higher than that of TCC, and the charge transfer resistance is reduced to 1.042 Ω. Barder charge shows that Cr(H_2_O)_5_Cl^2+^ has more electron transfer on its surface, and the change in charge density is more significant, thereby improving the reaction activity of Cr^3+^/Cr^2+^. In addition, in situ DEMS testing applied for the first time to the ICRFBs system showed that the hydrogen evolution reaction was greatly inhibited compared to the original TCC electrode. The mechanism was that H⁺ was more likely to adsorb on the surface of the In_2_O_3_ catalyst, and the binding energy barrier of H⁺ on its surface was higher, making it more difficult to occur H_2_ evolution reaction. It is interesting that the ICRFBs assembled with In_2_O_3_‐TCC electrodes also showed an EE of up to 84.02% and can be stably cycled 500 times. This work provides a profound understanding of the rational design of metal oxide catalyst‐modified electrodes to improve the performance of ICRFBs.

## 2 Results and Discussion

### 2.1 Material Synthesis and Characterization

The schematic diagram of the preparation of In‐based catalytic electrodes is shown in Figure 1e. The phase structure of the sample was studied using the X‐ray diffraction (XRD) method (Figure S2, Supporting Information). In the XRD pattern of the TCC electrode, there are two broad peaks at 25.5° and 43.4°, corresponding to the (002) and (100) crystal planes of C. The InOCl‐TCC samples grown in situ by the low‐temperature method showed typical peaks centered at 10.9°, 24.5°, 33.6°, and 44.6°, belonging to the (001), (101), (110), and (200) crystal planes of InOCl (PDF#11‐0510), respectively. The In_2_O_3_‐TCC samples grown in situ by the high‐temperature method showed typical peaks centered at 30.6°, 35.5°, and 51.0°, belonging to the (222), (400), and (440) crystal planes of In_2_O_3_ (PDF#06‐0416), respectively. The relative Rietveld refinement profiles show that the octahedral In_2_O_3_‐TCC sample prepared by direct high‐temperature solution thermal decomposition method has high phase purity (Figure 1c). However, spherical In_2_O_3_‐TCC prepared by treating InOCl‐TCC samples at the same high temperature showed broadening of peaks due to polycrystalline or small size effects, without preferred orientation (Figure 1d).

We also used Grazing Incidence Wide‐Angle X‐ray spectroscopy (GIWAXS) to study the surface structures of raw carbon cloth, sheet‐like InOCl catalytic layer prepared by low‐temperature solution thermal decomposition method, and octahedral In_2_O_3_ catalytic layer prepared by high‐temperature solution thermal decomposition method. The phase evolution is closely related to the temperature‐driven crystal structure transformation during the preparation process. As shown in Figure 1f, the 2D GIWAXS pattern shows that only the C (002) crystal plane can be observed in the TCC sample, while the InOCl‐TCC electrode exhibits distinct (101), (110), and (012) crystal planes. Under low temperature conditions, the In precursor undergoes local hydrolysis and coordination reactions on the surface of carbon cloth, with weaker interlayer forces on its (001) crystal plane, making it easier to spread and grow into a sheet‐like morphology along the carbon cloth. The In_2_O_3_‐TCC electrode exhibits distinct (222) and (400) crystal planes, indicating the formation of In_2_O_3_ phases. The direct high‐temperature solution thermal decomposition method achieves an ordered arrangement of In^3^⁺ through a one‐step reaction. The (222) crystal plane becomes the preferred growth direction due to its lower surface energy barrier, ultimately forming an octahedral structure with clear orientation. This is also the reason for its sharp XRD peak shape and high purity. The spherical In_2_O_3_‐TCC prepared by high‐temperature conversion of InOCl‐TCC, due to the sheet‐like structure of the precursor InOCl, restricts the atomic diffusion path, resulting in polycrystalline agglomeration or size non‐uniformity during the In^3^⁺ reconstruction process, leading to XRD peak broadening and no obvious orientation. The specific characterization of surface structure by GIWAXS (Figure S3, Supporting Information) further confirms that high‐temperature treatment can effectively promote the crystal phase transformation of In‐based compounds on the carbon cloth surface, and the direct high‐temperature method is more conducive to the exposure of highly active (222) crystal faces, laying a structural foundation for subsequent electrochemical performance optimization.

### 2.2 Morphological and Structural Characterizations

**Figure 2:**
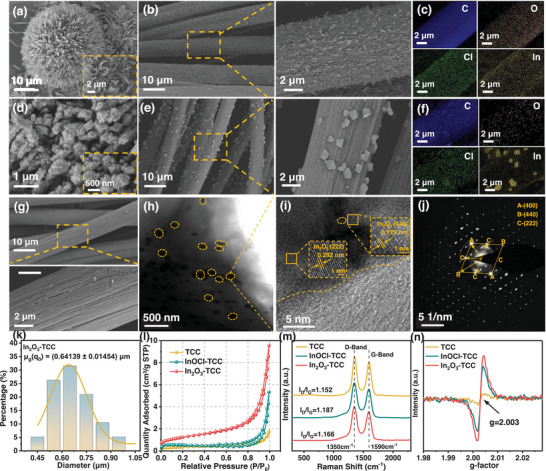
SEM images of a) InOCl and d) In_2_O_3_. SEM images of b) InOCl‐TCC and e) In_2_O_3_‐TCC electrodes. The EDS mapping of C, N, O, and In elements of the c) InOCl‐TCC and f) In_2_O_3_‐TCC electrodes. g) SEM images of the TCC electrode. h) TEM, i) HRTEM images, and j) the selected area electron diffraction (SAED) image of the In_2_O_3_‐TCC electrode. k) The particle size distribution diagram of octahedral In_2_O_3_. l) Nitrogen adsorption and desorption isotherms, and m) Raman spectra of the TCC, InOCl‐TCC, and In_2_O_3_‐TCC electrodes. n) EPR spectrum.

Figure 2a,d show scanning electron microscopy (SEM) images of InOCl prepared by reaction at 280 °C and In_2_O_3_ material prepared by reaction at 500 °C. It can be seen that the substrate is covered with large sheet‐like nanostructures, with a length of 3–4 µm and a width of 0.5–1.5 µm (**Figure** **2**a). Under heating conditions of 500 °C, the obtained sample is an octahedral structure with a size of ≈400 nm (Figure 2d). The surface of the heat‐treated carbon cloth is uneven, providing sufficient active sites for the growth of the catalyst (Figure 2g). The sample was successfully loaded onto the surface of carbon cloth using immersion and heat treatment methods. Figure 2b,e show carbon cloth samples of InOCl nanosheets (InOCl‐TCC) and In_2_O_3_ nanoparticles (In_2_O_3_‐TCC) grown in situ on the surface, respectively. It can be seen that the surface of InOCl‐TCC is uniformly distributed and has a dense sheet‐like structure. The length of the nanosheets is 0.3–0.7 µm, and the width is 0.1–0.3 µm (Figure S5b,c, Supporting Information), which is smaller than the size grown directly on the silicon wafer. This is because the partial binding with the carbon surface limits the production of nanosheets. The TCC carbon cloth uniformly loaded with InOCl nanosheets on the surface was further treated at 500 °C to obtain spherical indium oxide nanoparticles with a particle size of ≈60 nm (Figure S4, Supporting Information). However, uniform octahedral nanoparticles with a size of ≈650 nm can be observed on the surface of In_2_O_3_‐TCC prepared by direct heat treatment at 500 °C (Figure 2k).

Figure 2c,f show the EDS elemental mapping results of InOCl‐TCC and In_2_O_3_‐TCC electrodes, respectively. The distribution of C, O, Cl, and In on the carbon cloth surface indicates the successful growth of InOCl and In_2_O_3_. Then, the microstructure of In_2_O_3_ on the electrode was studied using transmission electron microscopy (TEM, Figure 2h). The lattice fringes of HRTEM can be clearly observed, with a lattice spacing of 0.179 and 0.292 nm, respectively, which is consistent with the distance between the (440) and (222) planes of octahedral In_2_O_3_ (Figure 2i). In addition, the selected region electron diffraction (SAED) pattern indicates that the surface is a single crystal of In_2_O_3_, and the diffraction patterns correspond to the (400), (440), and (222) planes of octahedral In_2_O_3_ (Figure 2j).

The wetting ability of the electrode is shown in Figure S6 (Supporting Information). The TCC electrode obtained after heat treatment has good hydrophilicity due to the increase of surface oxygen‐containing functional groups. When the three samples are gently placed on the surface of the electrolyte, they can all quickly be immersed in the solution. The contact angle test shows that the droplet can quickly penetrate into the three electrodes within 1 s. This indicates that the newly generated catalytic layer on the electrode surface does not reduce the high wettability of the TCC substrate.

Through N_2_ adsorption/desorption experiments, it can be observed that the modified electrode exhibits a significant hysteresis loop during the adsorption and desorption processes, belonging to the *I–V*‐type adsorption isotherm (Figure 2l). From Figure S7 (Supporting Information), it can be seen that the InOCl‐TCC electrode exhibits a higher BET specific surface area of 1.507 m^2^ g^−1^ than the TCC electrode (1.224 m^2^ g^−1^), while the In_2_O_3_‐TCC electrode has the highest data of 4.300 m^2^ g^−1^. From Figure S8 (Supporting Information), it can be seen that the number of mesopores in the In_2_O_3_‐TCC electrode is almost four times that of the TCC electrode. By in situ growth of InOCl and In_2_O_3_ catalyst on the TCC surface, mesopores were introduced to shorten the diffusion distance of ions on the electrode surface, increase the local flow rate of the electrolyte, promote rapid ion transfer, and enhance the diffusion of active ions on the electrode surface.^[^ ^41^ ^]^

The D peak at 1350 cm^−1^ in Raman spectroscopy represents lattice defects in carbon materials, while the G peak at 1590 cm^−1^ represents ordered graphite structures with sp2 hybridization (Figure 2m). The ratio of I_D_/I_G_ is commonly used to evaluate the degree of graphitization of carbon materials.^[^ ^42^ ^]^ I_D_/I_G_ ratios of the modified InOCl‐TCC and In_2_O_3_‐TCC electrodes are slightly increased compared to the TCC electrode, indicating an increase in the degree of defects in the modified electrode and a slight decrease in the degree of graphitization. Additionally, secondary calcination and surface catalyst loading had little effect on the graphitization degree and carbon structure of the carbon fiber surface.

To confirm the Ov in the indium‐based catalytic electrode, electron paramagnetic resonance (EPR) tests were performed on different electrode samples (Figure 2n). The characteristic signal at g = 2.003 originates from unpaired electrons captured by Ov in the In_2_O_3_ lattice. The absence of oxygen atoms forms defect sites with unoccupied orbitals, which can capture free electrons to form local magnetic moments and generate paramagnetic resonance signals in an external magnetic field. The strong signal of In_2_O_3_‐TCC indicates that its Ov concentration is significantly higher than TCC, which is closely related to the high‐temperature solution thermal decomposition process. Under high‐temperature conditions, the decomposition of In precursor is accompanied by the separation of O atoms, and high surface energy crystal faces are more prone to defects to reduce energy. The preferential growth of (222) crystal faces further promotes the formation of surface Ov. This Ov is not only an important source of active sites, but also carries local charges that can alter the surface electronic state of In_2_O_3_. By increasing the electron cloud density of In atoms, the adsorption capacity of Cr^3^⁺/Cr^2^⁺ is enhanced, while reducing the energy barrier during electron transfer, which will greatly improve the redox kinetics.

### 2.3 Atomic and Electronic Structural Characterization

**Figure 3:**
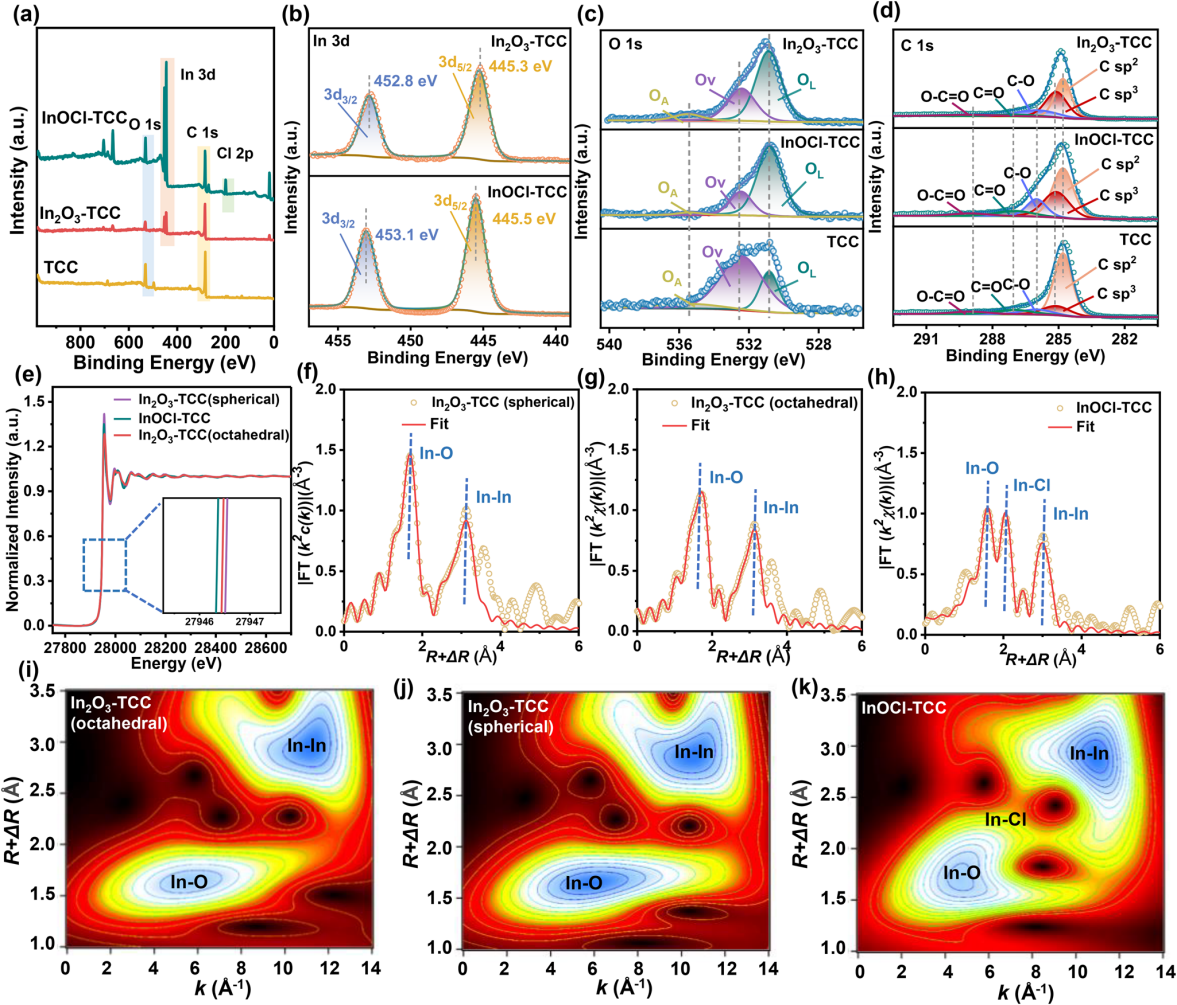
a) Summary of XPS spectra for electrodes. High‐resolution XPS spectra of b) In 3d for InOCl‐TCC and In_2_O_3_‐TCC electrodes. High‐resolution XPS spectra for electrodes of c) O 1s and d) C 1s. e) In K‐edge XANES spectra of the electrodes. EXAFS fitting curves of R space for f) In_2_O_3_‐TCC (spherical), g) In_2_O_3_‐TCC (octahedral), and h) InOCl‐TCC. Wavelet transform plots of i) In_2_O_3_‐TCC (spherical), j) In_2_O_3_‐TCC (octahedral), and k) InOCl‐TCC.

The surface composition and chemical state of different electrodes were further studied using X‐ray photoelectron spectroscopy (XPS). The XPS spectrum in **Figure** **3**a shows the presence of C and O elements in TCC, InOCl‐TCC, and In_2_O_3_‐TCC. An additional peak corresponding to Cl 2p was observed in InOCl‐TCC, with two different peaks located at 199.4 eV (Cl 2p_3/2_) and 200.9 eV (Cl 2p_1/2_), respectively (Figure S9, Supporting Information). The In 3d peak was observed in InOCl‐TCC and In_2_O_3_‐TCC samples (Figure 3b). The peaks of InOCl‐TCC (445.5 eV) and In_2_O_3_‐TCC (445.3 eV) belong to 3d_5/2_, with peak centers of ≈453.1 eV (InOCl‐TCC) and 452.8 eV (In_2_O_3_‐TCC) corresponding to 3d_3/2_. The shift of the indium characteristic peak of In_2_O_3_‐TCC reflects the interface electronic interaction with carbon cloth. These demonstrate the successful introduction of In and Cl elements into carbon materials, respectively. The C 1s spectrum was deconvoluted into C sp^2^ (284.8 eV), C sp^3^ (285.1 eV), C─O (286.0 eV), C═O (287.0 eV), and O─C═O (289.0 eV) (Figure 3d). From TCC to InOCl‐TCC and In_2_O_3_‐TCC electrodes, it can be observed that the original carbon (C sp^2^) content decreased from 62.75% to 44.35% and 39.85%, while the defect carbon (C sp^3^) content increased from 14.40% to 28.53% and 34.62%, indicating that the modified electrode can improve the reaction kinetics of the active material.

Meanwhile, the O 1s spectrum can be deconvoluted into three signals at 535.5, 532.4, and 530.8 eV (Figure 3c), corresponding to adsorbed oxygen (O_A_), oxygen vacancy (O_V_), and lattice oxygen (O_L_), respectively. As shown in Table S1 (Supporting Information), the InOCl‐TCC and In_2_O_3_‐TCC samples have higher oxygen functional group content, which helps to endow the electrode with superior hydrophilicity. At the same time, it can be observed that the Ov content decreased from 67.64% of the TCC to 22.33% of InOCl‐TCC and 31.25% of In_2_O_3_‐TCC. The core reason may be that the dominant type of surface oxygen species changed after the carbon cloth surface was covered by indium‐based compounds. The high Ov in primitive carbon cloth originates from the abundant surface defects and oxygen‐containing functional groups, which are characterized as “defect oxygen” signals. The InOCl‐TCC has a relatively regular crystal structure, a stable In─O bond, and fewer native Ov. Its load covers the defect sites of carbon cloth, making the surface oxygen species mainly “lattice oxygen” of indium‐based compounds, resulting in a significant decrease in the overall Ov ratio. This phenomenon indicates that indium‐based materials have effectively modified the carbon cloth surface, and the physical and chemical properties of subsequent materials are more likely to be dominated by the characteristics of InOCl/In_2_O_3_, rather than the defects of the original carbon cloth.

To accurately explore the local interface structure of In_2_O_3_‐TCC, the corresponding hard X‐ray absorption near‐edge structure (XANES) and extended X‐ray absorption fine structure (EXAFS) were analyzed. The detailed fitting parameters are listed in Table S2 (Supporting Information). As shown in the K‐edge XANES spectrum (Figure 3e), compared with spherical indium oxide, octahedral In_2_O_3_ shifts toward lower energy positions, and its valence state decreases. The absorption threshold position (blue dashed box) of InOCl is close to the position of In_2_O_3_. This indicates that the valence state of indium species in InOCl is approximately +3. In contrast, the part with the least electrons showed a peak intensity sequence of spherical In_2_O_3_ > InOCl> octahedral In_2_O_3_, indicating an increase in the number of electrons in octahedral In_2_O_3_ and enhanced electron‐donating ability, which is beneficial for Cr adsorption and activation.

The difference in coordination structure between In_2_O_3_ and InOCl revealed by Fourier Transform (FT) EXAFS spectrum is due to the crystal structure characteristics and defect control mechanisms of the materials, which are directly related to the electronic environment of their catalytic active sites. The fitting results in *R*‐space (Figure 3f,g) and k‐space (Figure S10, Supporting Information) for spherical In_2_O_3_ and octahedral In_2_O_3_ indicate that spherical In_2_O_3_ and octahedral In_2_O_3_ exhibit distinct peaks at 3.45 and 3.42 Å, respectively, which can be attributed to In‐In coordination species and to In─O coordination species at 2.17 Å. The coordination numbers (CN) of In─O and In─In in octahedral In_2_O_3_ are 5.8 and 5.7, respectively, which are lower than the In─O (6.0) and In─In (6.0) coordination numbers of spherical In_2_O_3_ samples, proving the existence of Ov. The formation of Ov leads to the loss of local O atoms, breaks the original coordination balance, and causes lattice microdistortion, resulting in a reduction of In‐In bond length from 3.45 to 3.42 Å. This distorted coordination environment due to the acceptance of electrons from unoccupied orbitals provided by Ov increases the electron cloud density of In atoms, optimizes their adsorption energy and electron transfer efficiency, and becomes a key structural basis for improving Cr^3^⁺/Cr^2^⁺ redox kinetics. In contrast, the In─O and In─In coordination numbers of spherical In_2_O_3_ remain at theoretical values (6.0), indicating a more complete lattice and fewer Ov, which is related to its polycrystalline disordered structure. Rapid agglomeration growth of spherical particles reduces the formation of surface defects, resulting in weaker control of the number of active sites and electronic states compared to octahedral In_2_O_3_. The peak of the InOCl sample at 2.04 Å is attributed to the In‐O coordinating species, the peak at 3.34 Å is attributed to the In─In coordinating species, and a new peak at 2.47 Å has a coordination number of 4.1, which can be attributed to the appearance of the In─Cl structure, confirming the presence of Cl^−^ in its layered structure. The Cl^−^ has a stronger electronegativity than O^2^^−^, which reduces the electron density of In atoms through electrostatic interactions, and the stability of In─Cl is lower than that of In─O bonds, making it difficult to provide effective active sites.

The signal difference of wavelet transform (WT) EXAFS (Figure 3i–k) further confirms the particularity of the coordination environment. The In─O and In─Cl bonds exhibit different intensity distributions in k‐space, and the heavy atom scattering signal of Cl is stronger in the high k region, directly distinguishing the essential difference between pure O coordination in In_2_O_3_ and mixed coordination in InOCl. The regulation of this coordination structure is the core mechanism for optimizing catalytic activity, providing atomic‐level evidence for understanding the structure‐activity relationship.

### 2.4 Improve Reaction Kinetics Performance

**Figure 4:**
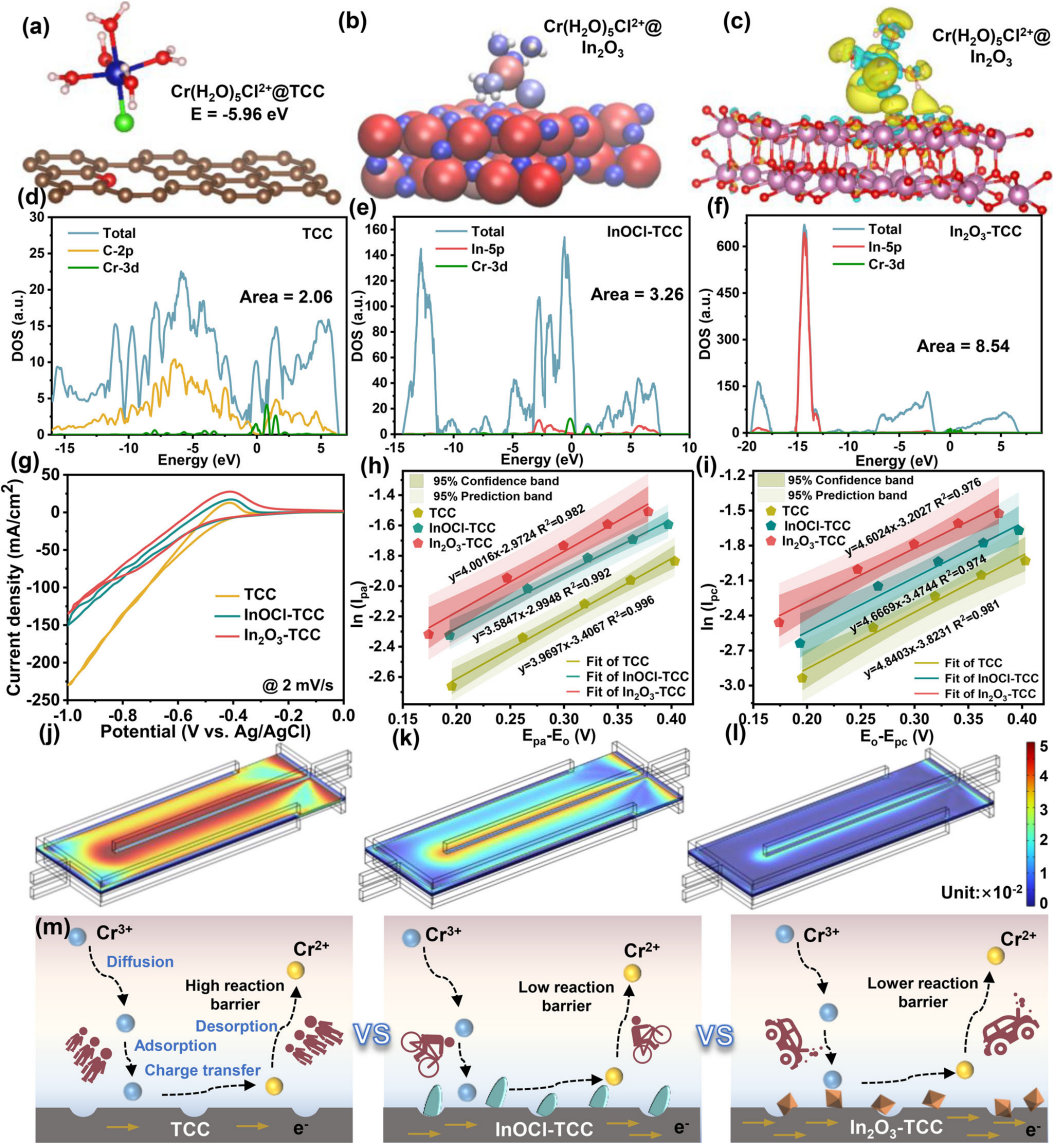
a) Adsorption energy and adsorption configuration of Cr(H_2_O)_5_Cl^2+^ on TCC surfaces. b) Bader charge of Cr(H_2_O)_5_Cl^2+^ adsorbed on In_2_O_3_ surfaces. c) The front views of the charge difference of Cr(H_2_O)_5_Cl^2+^ adsorbed on In_2_O_3_ surfaces. The density of states analysis of the Cr(H_2_O)_5_Cl^2+^ on d) TCC, e) InOCl, and f) In_2_O_3_ surfaces. g) CV curves of TCC, InOCl‐TCC, and In_2_O_3_‐TCC electrodes toward Cr^3+^/Cr^2+^ redox reaction at the scan rate of 2 mV s^−1^. The fitting lines of ln *Ip* and (*Ep‐Eo*) for h) Fe^2+^ oxidation and i) Fe^3+^ reduction processes on different electrodes. The distribution of overpotential in half‐cells with j)TCC, k) InOCl‐TCC, and l) In_2_O_3_‐TCC electrodes. m) Improvement mechanism for the redox kinetics by In_2_O_3_‐TCC.

Based on DFT mechanism calculations, we elucidated the potential mechanism of In_2_O_3_ promoting negative electrode Cr reaction activity and inhibiting HER at the atomic level. **Figure** **4**a and S11 (Supporting Information) respectively depict the adsorption energies of Cr(H_2_O)_5_Cl^2+^ on the surfaces of defective graphite (001), InOCl (101), and In_2_O_3_ (222). It can be seen that the adsorption energy of Cr(H_2_O)_5_Cl^2+^ on the surface of In_2_O_3_ (−11.87 eV) is significantly lower than that on the surfaces of TCC (−6.38 eV) and InOCl (−5.96 eV), indicating that Cr(H_2_O)_5_Cl^2+^ has a stronger adsorption ability on the surface of In_2_O_3_.

Calculate the Bader atomic charge of Cr(H_2_O)_5_Cl^2+^ on different surfaces and color the resulting atomic structure diagram. Blue represents a negative charge, indicating that the atom has gained electrons. And red represents a positive charge, indicating that the atom has lost electrons. Figure 4b is the adsorption structure of Cr(H_2_O)_5_Cl^2+^ molecules on the In_2_O_3_ surface and the total charge transfer amount is 1.26 |e|, which is higher than the total charge transfer amount on the TCC surface (0.35 |e|) and InOCl surface (1.18 |e|) (Figure S12, Supporting Information), indicating that Cr(H_2_O)_5_Cl^2+^ ions on the In_2_O_3_ catalyst surface exhibit higher redox activity.

From the differential charge diagram (Figure 4c; Figure S13, Supporting Information), it can be seen that compared with the TCC and InOCl surfaces, Cr(H_2_O)_5_Cl^2+^ adsorbed on the surface of the In_2_O_3_ catalyst has the highest charge transfer density, indicating that Cr(H_2_O)_5_Cl^2+^ has more electron transfer and higher reaction activity on the In_2_O_3_ catalyst surface. The density of states (DFT) calculation (Figure 4d–f) shows that the overlap area between Cr‐3d and In‐5p orbitals in In_2_O_3_ is 8.54, which is much larger than the overlap area between Cr‐3d and C‐2p (2.06) in the original defect graphite and Cr‐3d and In‐5p (3.26) in InOCl. The larger overlap area when Cr(H_2_O)_5_Cl^2+^ is adsorbed on the surface of the In_2_O_3_ catalyst indicates enhanced d‐p orbital hybridization, which is beneficial for charge transfer in the anode Cr^3+^/Cr^2+^ redox reaction. Besides, the reduction of Cr(H_2_O)_5_Cl^2+^ on In_2_O_3_ exhibited the lowest free energy change (ΔG≈−2.38 eV), confirming that the In_2_O_3_ catalyst enhanced the catalytic performance for Cr reaction activity (Figure S14, Supporting Information). Electronic local function (ELF) analysis was conducted on different surfaces (Figure S15, Supporting Information). It can be seen that in the adsorption configuration of In_2_O_3_ and Cr(H_2_O)_5_Cl^2+^, a significant high ELF value region appears between chromium and surface unsaturated O atoms, indicating the presence of a large and significant electron transfer from surface O to Cr, indicating that the In_2_O_3_ surface has the strongest catalytic promotion ability for Cr^3+^/Cr^2+^ redox reaction.

The electrochemical activity of different electrodes was tested by cyclic voltammetry (CV). Compared with the TCC, modified electrodes exhibit stronger performance in peak current density, peak separation, and peak current ratio, with In_2_O_3_‐TCC exhibiting the best performance. From the oxidation‐reduction reaction of Fe^2+^/Fe^3+^ in Figure S16a (Supporting Information) and the corresponding electrochemical data (Table S3, Supporting Information), it can be seen that the peak current densities of the oxidation and reduction of In_2_O_3_‐TCC are 98.4 and −85.5 mA cm^−2^, significantly higher than the TCC (69.9 and −52.9 mA cm^−2^) and InOCl‐TCC (97.5 and −71.4 mA cm^−2^). Moreover, the peak potential separation (∆Ep) of In_2_O_3_‐TCC decreased to 0.343 V compared to TCC (0.367 V), indicating its good reversibility and catalytic activity. In the CV curve of the Cr^3+^/Cr^2+^ redox reaction (Figure 4g), the peak current of the In_2_O_3_‐TCC electrode significantly increased, and the hydrogen evolution current density decreased, indicating that the In_2_O_3_ catalyst improved the activity of Cr^3+^/Cr^2+^ and suppressed the side reaction activity of hydrogen evolution.

We changed the scanning rate of CV testing from 2 to 10 mV s^−1^ to evaluate the mass transfer characteristics of different carbon cloth electrodes. It can be seen that as the scanning rate increases, the redox current density of the three samples gradually increases (Figure S16 and S18, Supporting Information). The results indicate that In_2_O_3_‐TCC can generate higher current density and smaller ∆Ep at all scan rates. The ‐Ipa/Ipc values obtained at each scanning speed indicate that among the three electrodes, the ‐Ipa/Ipc value of the In_2_O_3_‐TCC electrode is closer to 1.0, indicating superior reversibility and a more stable reaction (Figure S17a, Supporting Information). By evaluating the mass transfer characteristics through the relationship between the square root of peak current density and scanning rate,^[^ ^43^ ^]^ it can be seen that the redox reaction on the surface of carbon cloth is diffusion‐controlled (Figure S17b, Supporting Information). Among them, the slope of the In_2_O_3_‐TCC electrode is larger than that of both TCC and InOCl‐TCC electrodes, indicating that it has a faster mass transfer rate. In order to obtain more accurate values for comparing all these electrodes, the reaction rate constants (K°) of all oxidation and reduction reactions were calculated (Figure 4h,i). The K° values for the oxidation and reduction processes on the In_2_O_3_‐TCC electrode are 1.94 × 10^−3^ and 1.55 × 10^−3^ cm s^−1^, respectively, which are much larger than the corresponding values on the TCC electrode (1.26 × 10^−3^ and 0.83 × 10^−3^ cm s^−1^).

Based on the distribution characteristics of overpotential and local current density in COMSOL simulation results, the performance improvement effect of catalyst modification on ICRFBs can be analyzed from the following aspects. The original TCC electrode shows a higher overpotential, indicating significant activation polarization and ohmic polarization during charge transfer (Figure 4j). In the group of surface‐loaded indium catalysts, the overpotential decreased overall (Figure 4k,l). This change is due to the increase of active sites on the electrode surface, which provides more reaction interfaces through a high specific surface area, effectively reducing the energy barrier for charge transfer and thus reducing activation polarization. In addition, catalysts may weaken the effect of ohmic polarization by optimizing the electron transport path at the electrode‐electrolyte interface. It is worth noting that the overpotential distribution of the In_2_O_3_‐TCC electrode is more uniform (Figure 4l), indicating that surface loading of In_2_O_3_ enhances reaction kinetics and also improves the electrochemical stability of the electrode surface.

The local current density distribution of TCC exhibits a clear “point‐like concentration” feature, with high current density areas limited to only a few active sites (Figure S19a, Supporting Information). This uneven distribution can easily lead to local material corrosion and limit the overall output power. In contrast, the group with an added indium catalyst showed a significant increase in current density and an expanded distribution range. The peak current density of In_2_O_3_‐TCC reaches 220 mA·cm^−2^, and the highly active region covers over 60% of the electrode surface (Figure S19c, Supporting Information). From a microscopic perspective, the adsorption‐desorption process between the active sites on the catalyst surface and reactants is more efficient, accelerating the charge transfer step, and consistent with the increase in exchange current density j_0_ in the Butler‐Volmer equation. Although high current density may exacerbate reactant consumption, simulation results show that the introduction of the catalyst did not lead to drastic gradient changes in the concentration field, suggesting that the porous structure of the catalyst promotes mass transfer.

Different electrodes exhibit semicircles in the high‐frequency region and diagonal lines in the low‐frequency region at different frequencies of the reaction of Fe^2+^/Fe^3+^ and Cr^3+^/Cr^2+^(Figure S20, Supporting Information), corresponding to charge transfer processes and diffusion control processes, respectively. The corresponding equivalent circuit diagrams are shown in Figure S21 (Supporting Information). It can be seen that for the positive electrode reaction, the charge transfer resistance of In_2_O_3_‐TCC (Rct = 1.042 Ω) is lower than that of the TCC electrode (Rct = 2.534 Ω). For negative electrode reactions, the In_2_O_3_‐TCC electrode also has the smallest resistance value (Rct = 2.344 Ω) (Table S4, Supporting Information).

Figure S22 (Supporting Information) depicts the Tafel plots of TCC, InOCl‐TCC, and In_2_O_3_‐TCC electrodes to assess HER kinetics, a critical parasitic process in iron‐chromium redox flow batteries. The Tafel slope (a key indicator of HER kinetic barriers) is 70.88 mV dec^−1^ for TCC, 63.94 mV dec^−1^ for InOCl‐TCC, and 60.29 mV dec^−1^ for In_2_O_3_‐TCC. While a smaller Tafel slope typically suggests faster intrinsic HER dynamics, the role of In_2_O_3_‐TCC in suppressing HER arises from its ability to prioritize the target Cr^3^⁺/Cr^2^⁺ redox reaction. Specifically, In_2_O_3_ modification tailors the electrode surface to enhance Cr species adsorption and electron transfer, reducing the prevalence of H⁺‐involved HER. In contrast, TCC and InOCl‐TCC show weaker selectivity for Cr^3^⁺/Cr^2^⁺ redox, leading to more severe HER competition. Thus, In_2_O_3_‐TCC achieves HER suppression by favoring the desired redox process over parasitic HER. Figure S23 (Supporting Information) evaluated the overpotential of different electrodes in the electrolyte. It can be seen that as the current density increases, the overpotential also increases (Figure S23a, Supporting Information). At the current density of 120 mA cm^−2^, the overpotential of TCC is the highest (0.95 V), the overpotential of InOCl‐TCC relatively decreases (0.78 V), and the overpotential of In_2_O_3_‐TCC is the lowest (0.64 V). This indicates that the Cr^3+^/Cr^2+^ redox pair has higher electrochemical activity on the In_2_O_3_‐TCC electrode.

During battery cycling, the electrolyte may not reach all areas of the electrode surface. Eelectrochemically active surface area (ECSA) is used to accurately understand the accessible surface area of the electrolyte on the electrode surface.^[^ ^41^ ^]^ Increasing the scanning rate from 10 to 20 mV s^−1^, compared with TCC, the CV curve of In_2_O_3_‐TCC still maintains a rectangular shape without obvious polarization, indicating that the In_2_O_3_‐TCC sample has good reversibility and good rate performance^[^ ^44^ ^]^ (Figure S24, Supporting Information). Calculate the double‐layer capacitance (C_dl_) by linearly fitting the current variation (∆j) at −0.05 V with the scanning rate. The slope obtained by fitting the results with the least squares method shows that the slope of In_2_O_3_‐TCC (8.92 mF cm^−2^) is much larger than that of TCC (3.00 mF cm^−2^), indicating that it has a higher areal capacitance (Figure S24d, Supporting Information). These results indicate that the growth of In_2_O_3_ on the electrode fiber surface exposes more active sites, which is consistent with the physical characterization by SEM and BET. The catalytic advantage of In_2_O_3_‐TCC on the negative electrode Cr^3+^/Cr^2+^ pairs of ICRFBs was demonstrated (Figure 4m). Compared with TCC and InOCl‐TCC electrodes, the In_2_O_3_‐TCC electrode can effectively promote the redox reaction and rapid charge transfer of Cr ions on the electrode surface.

### 2.5 Inhibit Hydrogen Evolution Performance

**Figure 5:**
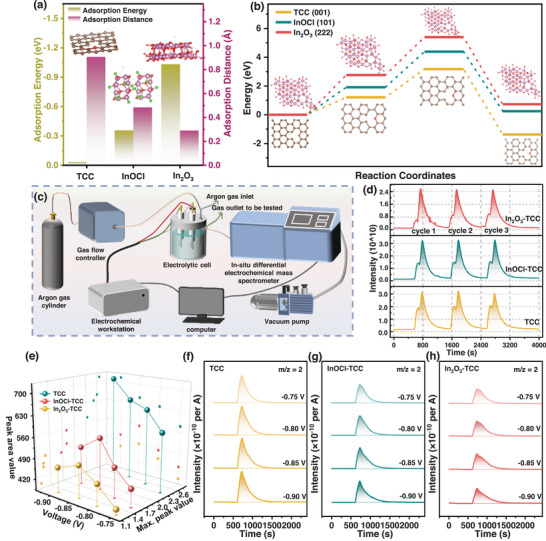
a) Adsorption energies of H^+^ on different surfaces. b) The activation energy barrier for HER on different surfaces. c) Schematic drawing of the DEMS setup and construction diagram of the specially designed DEMS. d) The mass signal results of m/z = 2 for DEMS cycling tests of different electrodes (each cycle is an LSV scan from −1.0 to 0.0 V vs. Ag/AgCl). f–h) DEMS data for CP method testing of TCC, InOCl‐TCC, and In_2_O_3_‐TCC electrodes at different voltages, and e) corresponding peak area and maximum peak height data.

The oxidation‐reduction potential of the Cr^3+^/Cr^2+^ pair in the ICRFBs is −0.41 V, which is close to the overpotential required for H^+^ to precipitate H_2_ on the surface of the carbon electrode. Therefore, the change in Cr activity will affect the hydrogen evolution reaction ability. Next, we investigated the inhibitory effect of modified electrodes on hydrogen evolution side reactions. The adsorption configurations and adsorption energy data of H^+^ on different surfaces in the electrolyte are shown in **Figure** **5**a. It can be seen that compared with the TCC and InOCl surfaces, the adsorption energy of H^+^ on the In_2_O_3_ surface is larger (−1.04 eV), and the adsorption distance is smaller (0.28 Å), which is more conducive to H^+^ obtaining electrons on the surface and becoming an adsorbed state. The transition state calculation method was used to simulate the migration process of H^+^ on three surfaces (Figure 5b; Figure S25, Supporting Information). It can be seen that the activation energy barrier for the hydrogen evolution reaction of H^+^ on the surface of In_2_O_3_ is higher, and H^+^ is more difficult to migrate and combine to generate H_2_ on the surface of the In_2_O_3_ catalyst, indicating that the In_2_O_3_ catalyst can effectively suppress hydrogen evolution reaction compared with TCC and InOCl.

To investigate the inhibitory effect of electrodes on HER, we designed a set of in situ differential electrochemical mass spectrometry (DEMS) for hydrogen evolution reactions of different electrodes in ICRFBs for the first time (Figure 5c). By conducting three cycles of LSV testing from −1 to 0 V on different electrodes (Figure 5d), the corresponding DEMS plots show that the hydrogen evolution curves of each cycle are almost identical, indicating that all three electrodes have good stability. The calculated Faraday efficiency (FE) and unit area hydrogen evolution rate of In_2_O_3_‐TCC electrode are 2.7% ± 0.3% and 0.11 µmol·cm^−2^h^−1^, respectively, which are much lower than those of TCC electrode at 18.5 ± 1.2% and 0.11 µmol·cm^−2^h^−1^. Figure S26 (Supporting Information) shows the corresponding hydrogen evolution peak area and maximum peak value. It can be seen that the amount of hydrogen evolution decreases from TCC to InOCl‐TCC and then to In_2_O_3_‐TCC, indicating that compared with TCC, InOCl‐TCC and In_2_O_3_‐TCC electrodes have the effect of suppressing HER, and In_2_O_3_‐TCC has the best effect. Figure 5f–h performed CP method testing for 100 s at different voltages ranging from −0.9 to −0.75 V to determine the hydrogen evolution rate and potential of different electrodes. From Figure 5e, it can be seen that the hydrogen evolution of InOCl‐TCC and In_2_O_3_‐TCC at different voltages is smaller than that of the TCC electrode, and the hydrogen evolution potential is shifted.

Figure S27 (Supporting Information) performed acid‐base titration on the anode electrolyte of ICRFB assembled with different electrodes after 80 cycles. It can be seen that after the same number of cycles, the concentration of H^+^ in the electrolyte of the battery assembled with In_2_O_3_‐TCC, InOCl‐TCC, and TCC electrodes decreased from 2.50 to 2.293, 1.820, and 1.656, respectively, reflecting the good inhibitory effect of In_2_O_3_‐TCC on HER (Table S5, Supporting Information). Figure S28 (Supporting Information) shows the linear sweep voltammetry (LSV) results of different electrodes. Compared to the Ag/AgCl reference electrode, the hydrogen evolution current density of the TCC electrode is significantly higher than that of other electrodes. This indicates that the presence of indium‐based catalysts effectively suppress H_2_ evolution, and the In_2_O_3_‐TCC electrode exhibits the lowest H_2_ evolution current density on its surface.

### 2.6 Redox Flow Battery Performance

**Figure 6:**
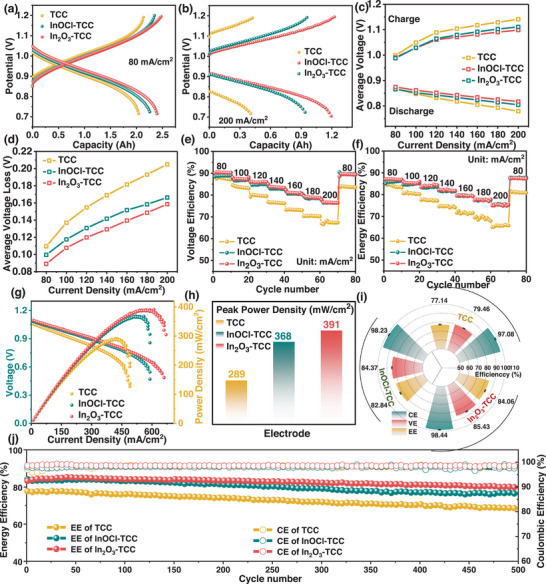
Charge–discharge curves of ICRFBs with different electrodes at a) 80 mA cm^−2^ and b) 200 mA cm^−2^. c) Average operation voltage and d) Average voltage loss with various current densities. e) VE and f) EE of ICRFBs with different electrodes with various current densities. The g) power density curves and h) corresponding peak power densities of ICRFBs with different electrodes. i) The CE, VE, and EE values of different electrodes at a current density of 140 mA cm^−2^. j) CE and EE in long‐term cycling with different electrodes at 140 mA cm^−2^.

To study the performance of the In_2_O_3_‐TCC electrode in actual ICRFBs, we constructed single cells based on different electrodes. Experiments were conducted on different electrodes at current densities of 80 and 200 mA cm^−2^ (**Figure** **6**a,b), and it was observed that the In_2_O_3_‐TCC electrode exhibited higher charging (2.42 Ah at 80 mA cm^−2^, 1.23 Ah at 200 mA cm^−2^) and discharging capacities (2.34 Ah at 80 mA cm^−2^, 1.19 Ah at 200 mA cm^−2^) and lower charging and discharging overpotentials. As the voltage of ICRFB is constantly changing during the cycling process, it is necessary to calculate the average voltage and its loss to comprehensively evaluate its performance (Figure 6c,d). The relative magnitude of charging and discharging voltage directly reflects the polarization of the battery and the performance of the electrodes. Among them, the charging voltage using In_2_O_3_‐TCC electrodes is the lowest, the discharging voltage is the highest, and the average voltage loss is smaller.

At a current density of 80–200 mA cm^−2^, the CE value slightly increases with the increase in current density (Figure S29, Supporting Information). At the same current density, due to the same membrane and electrolyte, the CE remains at the same value (Figure S30c, Supporting Information). The VE value is related to the overpotential of the battery and is directly affected by the surface modification of the electrode.^[^ ^45^ ^]^ The VE of batteries using InOCl‐TCC and In_2_O_3_‐TCC electrodes is higher than that of batteries using TCC at all current densities, resulting in higher EE (Figure 6e,f). At the current density of 200 mA cm^−2^, the VE and EE of the battery using the In_2_O_3_‐TCC (VE = 76.51%, EE = 75.41%) electrode are much higher than those of the TCC (VE = 67.33%, EE = 65.98%), respectively. When the current density is reset to 80 mA cm^−2^, its VE and EE values can be restored to their initial values. To evaluate the limitations of the power performance of ICRFBs, we conducted peak power density tests (Figure 6g). A typical polarization curve consists of three regions, mainly related to dynamic activation polarization at extremely low current density, ohmic polarization at medium current density, and concentration polarization at extremely high current density.^[^ ^46^ ^]^ Compared with the 289 mW cm^−2^ of TCC and the 368 mW cm^−2^ of InOCl‐TCC electrodes, the ICRFBs assembled with In_2_O_3_‐TCC electrodes exhibit higher peak power density (Figure 6h).

To evaluate the stability of the electrodes, we conducted 80 charge–discharge cycle tests at 140 mA cm^−2^. The average CE of the batteries assembled with the three electrodes was similar, but the average VE and EE of the batteries using In_2_O_3_‐TCC (EE = 84.02%) electrodes were much higher than those using InOCl‐TCC (EE = 82.92%) and TCC (EE = 77.26%) electrodes, and they also had higher capacity retention rates (Figure 6i; Figure S30, Supporting Information). In addition, we conducted the charge and discharge performance of ICRFBs with different electrodes for 500 cycles at 140 mA cm^−2^ (Figure 6j). The CE of the three batteries is almost the same, but the EE of the electrode using In_2_O_3_‐TCC is higher and more stable. After 500 cycles, the EE of the ICRFBs with TCC electrode decreased from 77.26% to 68.55%, and the EE of the ICRFBs with InOCl‐TCC electrode decreased from 82.92% to 76.04%. This capacity decay may be caused by the overlap of the pore size of the ion conduction channel formed by the sulfonic acid groups of the Nafion membrane used with the size of Fe^3^⁺ and Cr^2^⁺, which cannot effectively intercept metal ions. And the transmembrane H⁺ concentration difference induced by HER will further drive the permeation of positive and negative ions.^[^ ^47^ ^]^ The infiltrated Fe^3^⁺ will undergo a spontaneous redox side reaction with the negative electrode Cr^2^⁺, directly consuming the positive and negative electrode active materials, resulting in a decrease in the kinetics of the negative electrode Cr^3^⁺/Cr^2^⁺reaction, ultimately leading to capacity decay.

However, the ICRFBs using In_2_O_3_‐TCC electrodes exhibit high discharge capacity and electrolyte utilization (Figure S31, Supporting Information), with EE decreasing from 84.02% to 79.11%, demonstrating good catalytic performance and long cycle stability. Due to the dense crystal structure and high chemical bond energy of the prepared catalyst, it is not easy for the lattice to expand and break due to ion insertion/extraction during cycling. Therefore, only a few particles of the catalyst on the electrode surface after cycling have slight morphological changes, with very little reduction (Figure S32, Supporting Information).

## 3 Conclusion

In summary, through in situ high‐temperature thermal decomposition method, we have accurately constructed an octahedral structure In_2_O_3_ catalyst with exposed (222) crystal planes on the surface of carbon fibers (In_2_O_3_‐TCC), which has ideal performance in improving anode Cr^3+^/Cr^2+^ reaction kinetics and inhibiting hydrogen evolution. GIWAXS has demonstrated the successful preparation of In_2_O_3_. XANES indicates that octahedral In_2_O_3_ has oxygen vacancies that provide specific active sites, improving catalytic performance. Electrochemical tests showed that the cathode charge transfer resistance of the In_2_O_3_‐TCC electrode decreased to 1.042 Ω, the adsorption energy with Cr(H_2_O)_5_Cl^2+^ was stronger (−11.87 eV), the electron transfer efficiency of Cr(H_2_O)_5_Cl^2+^ on its surface was faster, and the anode Cr^3+^/Cr^2+^ reaction kinetics were enhanced. In addition, compared with the TCC surface, the adsorption energy (−1.04 eV) of H⁺ on the In_2_O_3_ anode catalyst surface is greater, and the binding energy barrier for the migration and binding of H^+^ adsorbed on the anode surface to generate H_2_ is higher, thereby suppressing the occurrence of anode HER. The assembled ICRFBs have a peak power density of up to 391 mW cm^−2^ and an EE of up to 84.02% at a current density of 140 mA cm^−2^. This work provides an effective approach for constructing ICRFBs with high power density, high EE, and long lifespan based on electrode design, accelerating their further demonstration applications. It is worth noting that this study focuses on exploring the basic mechanisms rather than direct industrial applications. The challenges of large‐scale deployment, such as optimizing indium resource supply and recovery strategies, further reducing thermal decomposition energy consumption, and verifying performance over thousands of cycles, require further research in the future. Overall, our research findings provide a mechanistic basis for designing high‐performance anode catalysts and accelerate the development of ICRFB toward practical demonstrations.

## Conflict of Interest

The authors declare no conflict of interest.

## Supporting information



Supporting Information

## Data Availability

The data that support the findings of this study are available from the corresponding author upon reasonable request.
