# Supplementary material for: Strategic Facet Design of In2O3 Catalysts for Enhanced Kinetics and Hydrogen Suppression in Iron–Chromium Flow Batteries
Source: Adv Sci (Weinh). 2025 Oct 8;13(2):e12148. doi: 10.1002/advs.202512148 (PMC12786334; doi:10.1002/advs.202512148)
Supplement: Supplementary file 1 — Supporting Information [file ADVS-13-e12148-s001.docx]

Supporting Information

Strategic Facet Design of In₂O₃ Catalysts for Enhanced Kinetics and Hydrogen Suppression in Iron–Chromium Flow Batteries

*Yinping Liu,^a#^ Chao Guo,^a#^ Fangang Qu,^a^ Yida Zhang,^b^* *Kuo-Wei Huang,^c^ Chunming Xu,^a^ Jia Guo,*^d^ Quan Xu,***^a^ Yingchun Niu,*^a^*

^a.^ State Key Laboratory of Heavy Oil Processing, China University of Petroleum (Beijing), Beijing 102249, China

^b.^ College of Chemical Engineering, Inner Mongolia University of Technology, Hohhot 010051, China

^c.^ Chemistry Program, Division of Physical Science and Engineering, King Abdullah University of Science and Technology (KAUST), Thuwal 23955-6900, Saudi Arabia

^d.^ Department of Chemistry, Aarhus University, 8200 Aarhus N, Denmark

**^#^** These authors contributed equally to this work.

**Experimental section**

**Materials**

Pristine carbon cloth (CC, the thickness is 0.8 mm) was purchased from Liaoning Jingu Carbide Co., Ltd. Hydrochloric Acid (HCl) was purchased from Sinopharm Chemical Reagent Co., Ltd. Ferrous chloride (FeCl_2_·H_2_O, AR) was purchased from Xilong Science Co., Ltd. The analytical grade chromium chloride (CrCl_3_·6H_2_O, 99%) and indium chloride (InCl_3_·4H_2_O, 99%) were purchased from Shanghai Macklin Biochemical Technology and Shanghai Aladdin Biochemical Technology. For all the experiments, deionized water was prepared by ourselves in the laboratory with an ultrapure purification system.

**Preparation of electrodes**

**Preparation of thermally treated carbon cloth (TCC) electrode:** The pristine carbon cloth (CC) was washed with ultrasound in deionized water for 15 minutes to avoid the influence of impurities and dried in a 60 ^o^C oven for later use. The dried carbon cloth was heated with a heating rate of 20 ^o^C/min in a 500 ^o^C constant temperature muffle furnace for 5 h in air atmosphere, then cooled to room temperature to form a thermally treated carbon cloth (TCC) electrode.

**Preparation of InOCl-modified thermally treated carbon cloth (****InOCl-TCC) electrode:** 0.0128g of InCl_3_·4H_2_O was dissolved in 10 mL of a solution containing 0.1 M hydrochloric acid to obtain an InCl_3_ aqueous solution. The TCC (size: 2 ×5 cm^2^) was soaked into the prepared InCl_3_ solution for 5 hours. Then the solution containing TCC was transferred to a muffle furnace, raised the temperature to 280 ^o^C at a rate of 20 ^o^C /min, and reacted for 2.5 hours. After natural cooling to room temperature, the InOCl-TCC electrode (0.5 mg/cm^2^) was obtained.

**Preparation of In_2_O_3_-modified heat-treated carbon cloth (In_2_O_3_-TCC) electrode (spherical In_2_O_3_-TCC mentioned in the article):** The InOCl-TCC electrode prepared above was placed in a muffle furnace, raise it to 500 °C at a rate of 5 °C/min, and maintain it at 500 °C for 1 hour. Afterwards, it was naturally cooled to room temperature to obtain a spherical In_2_O_3_-TCC electrode (0.5 mg/cm^2^).

**Preparation of** **In_2_O_3_-modified heat-treated carbon cloth (In_2_O_3_-TCC) electrode (octahedral In_2_O_3_-TCC mentioned in the article):** 0.0128g of InCl_3_·4H_2_O was dissolved in 10 mL of a solution containing 0.1 M hydrochloric acid to obtain the InCl_3_ aqueous solution, respectively. The TCC (size: 2 ×5 cm^2^) was soaked into the prepared InCl_3_ solution for 5 hours. Then the solution containing TCC was transferred to a muffle furnace, raised the temperature to 500 ^o^C at a rate of 20 ^o^C/min, and reacted for 2.5 hours. After natural cooling to room temperature, the In_2_O_3_-TCC electrode (0.5 mg/cm^2^) was obtained.

**Materials Characterization**

The micro morphologies of the materials were analyzed by scanning electron microscope (Sigma 500, Carl Zeiss (Shanghai) Management Co., Ltd) with energy dispersive spectroscopy (EDS) and transmission electron microscopy (TEM, FEI Tecnai G2 F30). X-ray photoelectron spectroscopy (XPS, ESCALAB250Xi, VG, UK) was performed to investigate the surface element composition and specific bonding state. The grazing incidence wide angle X-ray scattering (GIWAXS) measurements were performed at BL14B1 beamline, Shanghai Synchrotron Radiation Facility (SSRF). The wavelength of the X-ray was 1.24 A˚ (10 KeV) with an exposure time of 30s. The composition and crystal structure of the photocatalyst was explored by X-ray diffraction (XRD) spectroscopy (D2 PHASER, AXS AG, Brooke, Germany) via Cu Kα radiation in the 2θ range of 5-90^o^ at a scanning rate of 5^o^ min^-1^. The surface area and pore size distribution were analyzed using N_2_ adsorption/desorption (BET, MicroActive for ASAP 2460, Micro, America). The surface area was calculated through the Branauer-Emmett-Teller (BET) equation, and the pore size distribution was determined by the Barrett-Joiner-Helenda (BJH) method. O vacancies were detected using electron paramagnetic resonance (EPR), employing the Bruker A300-10/12 instrument. Hard X-ray absorption spectroscopy measurements were conducted at the In K edge (E_0_=27940 eV) on the beamline BL14W1 of a 3.5 GeV Shanghai Synchrotron Radiation Facility (SSRF). Athena and Artemis code are used to extract and fit data. For the X-ray absorption near edge structure (XANES) section, the experimental absorption coefficients as a function of energy µ(E) were processed using background subtraction and normalization procedures. For the extended X-ray absorption fine structure (EXAFS) section, the Fourier transformed data was fitted in R space. Perform wavelet transform analysis using lgor pro script developed by Funke et al.

**Electrochemical measurement**

Electrochemical properties of different electrodes were investigated by the electrochemical workstation (CHI 760E, Chenhua Instrument Co. Ltd., China) using a three-electrode setup with the carbon cloth electrode (1 cm^2^, TCC, InOCl-TCC, and In_2_O_3_-TCC, respectively) as working electrode, platinum plate as the counter electrode, and Ag/AgCl in saturated potassium chloride solution as the reference electrode. Both cathodic and anodic detections were performed using electrolytes with concentrations of 1.2 M FeCl_2_·4H_2_O + 1.4 M CrCl_3_·6H_2_O + 2.5 M HCl. The cyclic voltammetry (CV) was measured at different scan rates with voltage windows of 0 V to 1 V and −1 V to 0 V relative to Fe^2+^/Fe^3+^ and Cr^3+^/Cr^2+^ redox reactions. The electrochemical impedance spectroscopy (EIS) was tested by applying a 10 mV alternating voltage within the frequency range of 100 kHz to 0.1 Hz. In addition, the Tafel slopes (b) were evolved from LSV curves based on the Tafel equation (η = a + b log|j|, where a, j, and η are the intercept, current density, and over-potential, respectively). The double-layer capacitance values (C_dl_) were determined by CV curves from -0.1 to 0 V at various scan rates (10-20 mV/s).

**Calculation methods**

The adsorption energy and diffusion energy barrier were calculated by VASP software based on density functional theory (DFT). The generalized gradient approximation (GGA) of Perdew-Burke-Ernzerhof (PBE) is applied to depict the exchange-correlation function. The cut-off energy of the plane wave base is 450 eV. In geometric optimization, the energy convergence condition is 10^-6^, and the force convergence condition is set to 0.05 eV/Å. A 3 × 3 × 1 supercell containing 35 atoms was built for a single layer of C (001) surfaces, a 2 × 2 × 1 supercell containing 48 atoms was built for two layers of InOCl (101) surfaces and a 1 × 1 × 1 supercell containing 80 atoms was built for a two layer of In_2_O_3_ (222) surfaces were constructed. To avoid the dipole moment effect caused by interlayer interactions and periodic boundary conditions, the thickness of the vacuum layer along the Z-axis direction is set to 15 Å. The k-point meshes in the Mokhorst-Pack scheme were set to be 2×2×1 for surfaces.

This system is a hydrochloric acid system, and the concentration of chloride ions has a significant impact on the stability of chromium ion hydrates. There is a phenomenon where H_2_O molecules are replaced and then shed, and chloride ions bond with chromium ions in the solution, resulting in the formation of chromium chloride hydrates. Based on this actual situation, structural relaxation was carried out again in the presence of both chromium ion hydrates and chloride ions, resulting in a model of Cr(H_2_O)_5_Cl^2+^. The stable configuration obtained by structural self consistency was cubic hexagonal. The adsorption energy of Cr(H_2_O)_5_Cl^2+^ and H^+^ on the electrode surface E_ads_ was calculated by E_ads_ = E_total_ – E_cluster_ - E_surf_, where E_total_ and E_surf_ were the total energies of the electrode surface with and without Cr(H_2_O)_5_Cl^2+^ or H^+^ adsorbed respectively, and E_cluster_ refers to the energy that adsorbs the substance. To determine the thermodynamic properties of the adsorption process of Cr(H_2_O)_5_Cl^2+^ on TCC, InOCl, and In_2_O_3_ surface, we performed the frequency analysis on the adsorption configurations of Cr(H_2_O)_5_Cl^2+^ on the three materials, with an energy convergence criterion of 1 × 10^-6^ eV. Multi physics process simulation was conducted using COMSOL tool to establish a 10 cm^2^ ICRFB single cell model including electrodes and channels, coupling the interactions of multiple physical processes.

**Online DEMS measurements**

Differential Electrochemical Mass Spectrometry (DEMS, Shanghai Linglu Instrument Equipment Co., Ltd). During the measurement process of DEMS, LSV is scanned cyclically between the potential of -1 V and 0 V at a scanning rate of 4mV/s, and the signal intensity is recorded by a mass spectrometer. The Ag/AgCl is used as the reference electrode and 1 cm^2^ carbon cloth as the working electrode. Perform a 100-second chronopotentiometric (CP) test using the voltages of -0.9 V, -0.85 V, -0.80 V, and -0.70 V, respectively. Before online detection, the equipment is continuously supplied with argon gas for 20 minutes to exhaust the air in the pipeline.

Faraday efficiency (FE) calculation of hydrogen evolution reaction:

${FE}_{HER}=\frac{2\times n_{H2}\times F}{Q_{total}}\times100\%$ (1)

Where F=96485 C/mol is Faraday's constant, n_H2_ is the molar amount of H₂ measured by DEMS, and Q_total_ is the total charge.

Hydrogen evolution rate per unit area:

$r=\frac{n_{H2}}{A\times t}$ (2)

Where A is the electrode area and t is the testing time.

**Flow-battery measurement**

The performance of the carbon cloth electrodes in the ICRFBs cell was evaluated using the NEWARE Battery Test System (CT-4008T-5V12A-S1-F, Shenzhen, China). An ICRFB single cell was prepared by stacking the current collectors, bipolar plates, gaskets, flow frames, membranes, and carbon cloth electrodes. The ICRFBs were assembled with 1.2 M FeCl_2_·4H_2_O + 1.4 M CrCl_3_·6H_2_O + 2.5 M HCl as positive and negative electrolytes. The volume of both positive and negative electrolytes was 80 mL. A Nafion 212 membrane was used as the polymer electrolyte membrane. The TCC, InOCl-TCC, and In_2_O_3_-TCC electrodes with an active area of 10 cm^2^ (2×5 cm) were used as the positive and negative electrodes, respectively. During the rate and cyclic performance testing, the flow rate of the electrolytes at both negative and positive sides was 20 mL/min. The battery was operated in galvanostatic mode by changing current densities from 80 mA/cm^2^ to 200 mA/cm^2^. To protect the battery from being overcharged and overdischarged, the lower and upper cut-off voltages were preset to be 0.7 V and 1.2 V, respectively. The calculation formulas for Coulomb efficiency (CE), voltage efficiency (VE), energy efficiency (EE), and charge/discharge capacity performance of ICRFB are as follows.

Coulomb Efficiency (CE) = $\frac{Qdischarge}{Qcharge}\times100\%$ (3)

In the equations above, Qdischarge and Qcharge stand for discharge capacity (mAh/L) and charge capacity (mAh/L), respectively.

Voltage Efficiency (VE) = $\frac{Vdischarge}{Vcharge}\times100\%$ (4)

In the equations above, Vdischarge and Vcharge represent discharge voltage (V) and charge voltage (V), respectively.

Energy efficiency (EE) = Coulomb Efficiency (CE) $\times$Voltage Efficiency (VE) (5)

Charge capacity (Ah) = Charge current (A) $\times$Charge time (h) (6)

Discharge capacity (Ah) = Discharge current (A) $\times$Discharge time (h) (7)

**Economic calculation**

The cost structure of iron-based flow battery systems mainly consists of electrodes, ion exchange membranes, electrolyte, and control system. Based on a single kW iron-based flow battery system, combined with the iron-based system cost structure reported by Huan et al. [1] and the data from this study, the specific calculation is as follows:

Total system cost: Referring to the average bid price of the liquid flow battery energy storage system industry in 2024 (approximately 0.31 USD/Wh), the total cost of a single kW system is approximately 310 USD (assuming 1 kW=1 kWh energy storage capacity, the proportion of capacity cost is higher in long-term energy storage scenarios); In_2_O_3_-TCC catalyst cost: loading capacity of 0.5 mg/cm^2^, single kW electrode area of 2000 cm^2^, catalyst cost of 0.27 USD/g, then the total cost of single kW catalyst is 0.27 USD; Cost proportion: The proportion of In_2_O_3_-TCC catalyst in the total system cost is only 0.087%, far lower than the reported Bi modified catalyst and commercial carbon based catalyst, and even lower than the cost of electrolyte impurity control.

At the same time, under 140 mA/cm^2^ and a charge discharge cut-off voltage of 0.7~1.2 V, In_2_O_3_-TCC helps the entire battery achieve a high energy efficiency of 84.02% and an EE retention rate of over 79% after 500 cycles, reducing ineffective electrolyte consumption and replacement frequency, further reducing long-term operation and maintenance costs, and fully meeting the commercial core requirements of low-cost and long-life iron-based flow batteries.

**Fig. S1.** The density of states analysis of the Cr(H_2_O)_5_Cl^2+^ on (a) In_2_O_3_ (211), (b) In_2_O_3_ (400) and (c) In_2_O_3_ (440) surfaces.


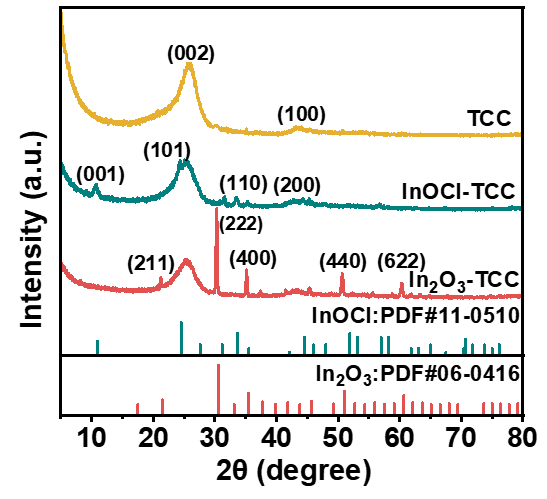


**Fig. S2.** X-ray diffraction pattern of the TCC, InOCl-TCC, and In_2_O_3_-TCC electrodes.

**Fig. S3.** (a, b) Radial integration corresponding GIWAXS patterns of the TCC, InOCl-TCC, and In_2_O_3_-TCC electrodes. (c) GIWAXS patterns and (d) radial integration of the In_2_O_3_-TCC (spherical) electrode.

**Fig. S4**. SEM images of the spherical In_2_O_3_-TCC prepared by the InOCl-TCC electrode.

**Fig. S5**. (a) The particle size distribution diagram of spherical In_2_O_3_. (b) The height and (c) width distribution diagram of the InOCl.

**Fig. S6.** Contact angles of the TCC, InOCl-TCC, and In_2_O_3_-TCC electrodes.

**Fig. S7.** BET surface area of TCC, InOCl-TCC, and In_2_O_3_-TCC electrodes.

**Fig. S8.** (a) Pore-size distribution curves and (b) Cumulative pore volume curve of TCC, InOCl-TCC, and In_2_O_3_-TCC electrodes.


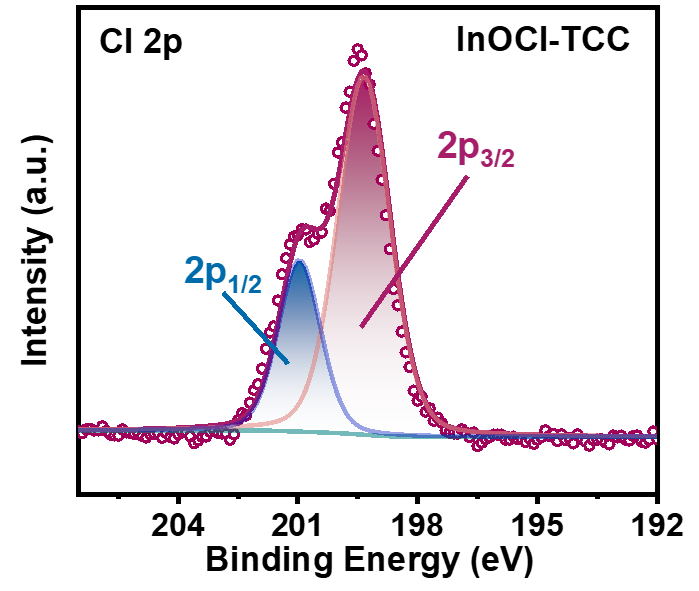


**Fig. S9.** High-resolution XPS spectra of Cl 2p for InOCl-TCC electrode

**Table S1.** Atomic fractions of C, O, In and Cl from XPS spectra of the electrodes.

|  | TCC | InOCl-TCC | In_2_O_3_-TCC |
| --- | --- | --- | --- |
| C | 95.48 | 85.16 | 84.80 |
| O | 4.52 | 10.08 | 12.62 |
| In | / | 2.42 | 2.58 |
| Cl | / | 2.34 | / |
| C1 | 62.75 | 44.35 | 39.85 |
| C2 | 14.40 | 28.53 | 34.62 |
| C3 | 8.60 | 14.63 | 16.42 |
| C4 | 9.08 | 8.40 | 4.60 |
| C5 | 5.17 | 4.09 | 4.51 |
| O1 | 22.67 | 74.98 | 62.56 |
| O2 | 67.64 | 22.33 | 31.25 |
| O3 | 9.67 | 2.69 | 6.19 |
| In1 | / | 39.19 | 40.25 |
| In2 | / | 60.81 | 59.75 |

**Fig. S10.** EXAFS fitting curves of k space for (a) In_2_O_3_-TCC (octahedral), (b) In_2_O_3_-TCC (spherical) and (c) InOCl-TCC.

**Table S2.** EXAFS fitting parameters at the In K-edge for various samples (*Ѕ*_0_^2^=0.85).

| Sample | Shell | *N^a^* | *R*(Å)*^b^* | *σ*^2^(Å^2^)*^c^* | Δ*E*_0_ (eV)*^d^* | *R* factor |
| --- | --- | --- | --- | --- | --- | --- |
| In_2_O_3_-TCC (spherical) | In-O | 6.0 | 2.17 | 0.0012 | 4.6 | 0.0033 |
|  | In-In | 6.0 | 3.45 | 0.0018 |  |  |
| In_2_O_3_-TCC (octahedral) | In-O | 5.8 | 2.17 | 0.0042 | 5.2 | 0.0012 |
|  | In-In | 5.7 | 3.42 | 0.0053 |  |  |
| InClO-TCC | In-O | 2.1 | 2.04 | 0.0015 | -3.6 | 0.0054 |
|  | In-Cl | 4.1 | 2.47 | 0.0010 |  |  |
|  | In-In | 6.0 | 3.34 | 0.0067 |  |  |

*^a^N*: coordination numbers; *^b^R*: bond distance; *^c^σ*^2^: Debye-Waller factors; *^d^* Δ*E*_0_: the inner potential correction. *R* factor: goodness of fit.

**Fig. S11.** Adsorption energy and adsorption configuration of Cr(H_2_O)_5_Cl^2+^ on (a) InOCl and (b) In_2_O_3_ surfaces.


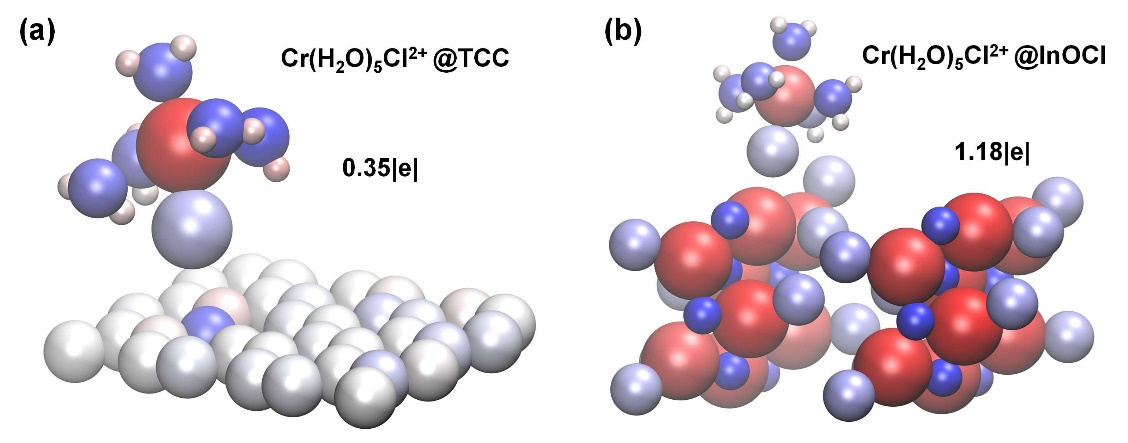


**Fig. S12.** Bader charge of Cr(H_2_O)_5_Cl^2+^ adsorbed on the (a) TCC and (b) InOCl surfaces.

**Fig. S13.** The top, front, and side views of the charge difference of Cr(H_2_O)_5_Cl^2+^ adsorbed on the surface of TCC, InOCl, and In_2_O_3_.


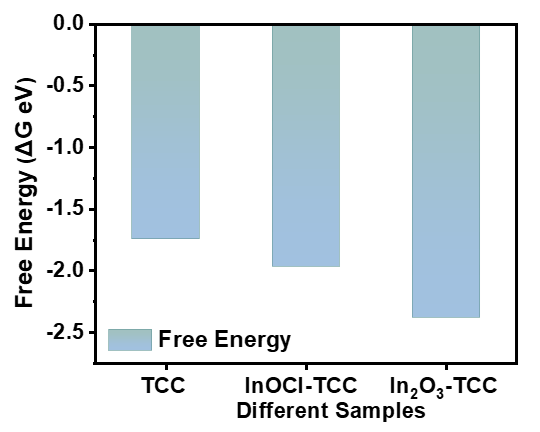


**Fig. S14.** The change of free energy from Cr^3+^ to Cr^2+^ with TCC, InOCl, and In_2_O_3_.

**Fig S15.** Electron localization function diagrams of different adsorption configurations of Cr(H_2_O)_5_Cl^2+^ with a) TCC, b) InOCl, and c) In_2_O_3_, respectively.

**Fig. S16.** (a) CV curves of TCC, InOCl-TCC, and In_2_O_3_-TCC electrodes toward Fe^2+^/Fe^3+^ redox reaction at the scan rate of 2 mV/s. CV curves of Fe^2+^/Fe^3+^ redox pairs at different scan rates for (b) TCC, (c) InOCl-TCC and (d) In_2_O_3_-TCC electrodes.

**Fig. S17.** (a) -Ipc/Ipa values versus different scan rates for the Fe^2+^/Fe^3+^ redox pairs. (b) Plots of the oxidation and reduction peak current versus the square root of scanning rate for different electrodes, respectively.

**Table S3.** Electrochemical data was obtained from CV curves of the electrodes for positive reactions at a scan rate of 2 mV/s.

| Electrode | Ipa (mA) | -Ipc (mA) | Epa (V) | Epc (V) | -Ipc/Ipa | ∆E (V) |
| --- | --- | --- | --- | --- | --- | --- |
| TCC | 69.9 | 52.9 | 0.635 | 0.268 | 0.757 | 0.367 |
| InOCl-TCC | 97.5 | 71.4 | 0.644 | 0.249 | 0.732 | 0.395 |
| In_2_O_3_-TCC | 98.4 | 85.5 | 0.657 | 0.314 | 0.869 | 0.343 |

**Fig. S18.** CV curves of Cr^2+^/Cr^3+^ redox pairs at different scan rates for (a) TCC, (b) InOCl-TCC and (c) In_2_O_3_-TCC electrodes.

**Fig. S19.** The distribution of local current density in half-cells with (a) TCC, (b) InOCl-TCC and (c) In_2_O_3_-TCC electrodes.

**Fig. S20.** EIS curves of (a) Fe^2+^/Fe^3+^ and (b) Cr^3+^/Cr^2+^ redox couples.

**Fig. S21.** Equivalent circuit model.

**Table S4.** Parameters fitted by Zview of the electrodes.

| Electrode | η(V) | Rs (ohm) | Rct (ohm) | η(V) | Rs (ohm) | Rct (ohm) |
| --- | --- | --- | --- | --- | --- | --- |
| TCC | 0.5 | 0.317 | 2.534 | -0.4 | 2.463 | 7.535 |
| InOCl-TCC | 0.5 | 0.191 | 1.380 | -0.4 | 1.649 | 4.055 |
| In_2_O_3_-TCC | 0.5 | 0.142 | 1.042 | -0.4 | 1.332 | 2.344 |


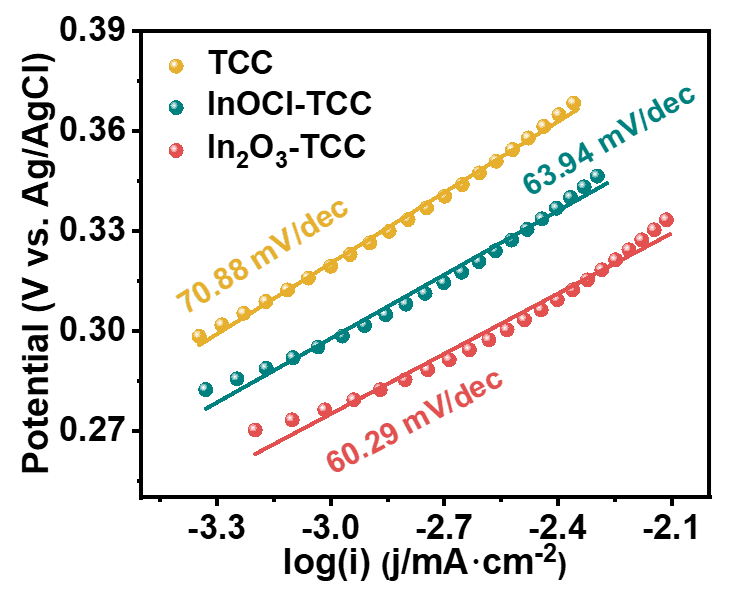


**Fig. S22.** Tafel plots of TCC, InOCl-TCC, and In_2_O_3_-TCC electrodes.

**Fig. S2****3.** Voltage-time test curves of different current density for (a) TCC, (b) InOCl-TCC and (c) In_2_O_3_-TCC electrodes.

**Fig. S24.** CV curves at different scan rates of the a) TCC, b) InOCl-TCC, and c) In_2_O_3_-TCC electrode. (d) Cdl of the TCC, InOCl-TCC and In_2_O_3_-TCC electrode.

**Fig. S25.** Minimal reaction energy pathway for hydrogen evolution at InOCl (101) surface.


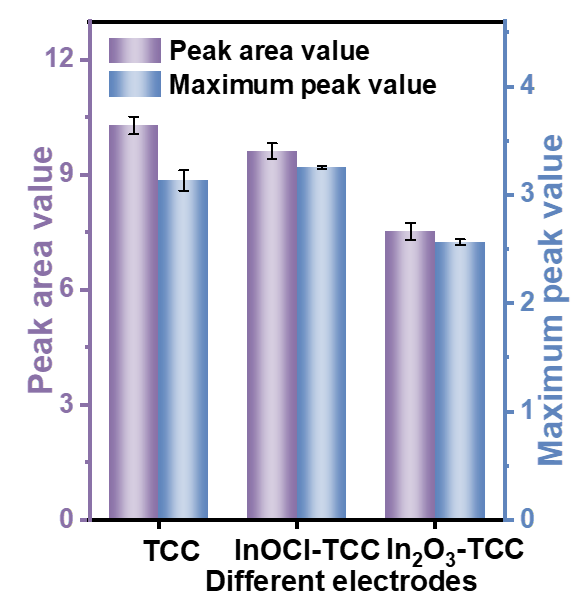


**Fig. S26.** The corresponding peak area and maximum peak height data.

**Fig. S27.** (a) The variation of pH value and (b) the trend of pH value changing with sodium hydroxide (NaOH) content during acid-base titration of negative electrode electrolyte after 80 cycles of ICRFBs assembled with different electrodes.

**Table S5.** Experimental parameters for acid-base titration of electrolytes after cycling of ICRFBs assembled with different electrodes.

| Samples | C(NaOH)/(mol/L) | d(pH)/dVmax~V(NaOH) | C(H^+^)/(mol/L) | ∆C(H^+^) |
| --- | --- | --- | --- | --- |
| TCC | 0.91 | 9.1 | 1.656 | 0.844 |
| InOCl-TCC | 0.91 | 10.0 | 1.820 | 0.680 |
| In_2_O_3_-TCC | 0.91 | 12.6 | 2.293 | 0.207 |


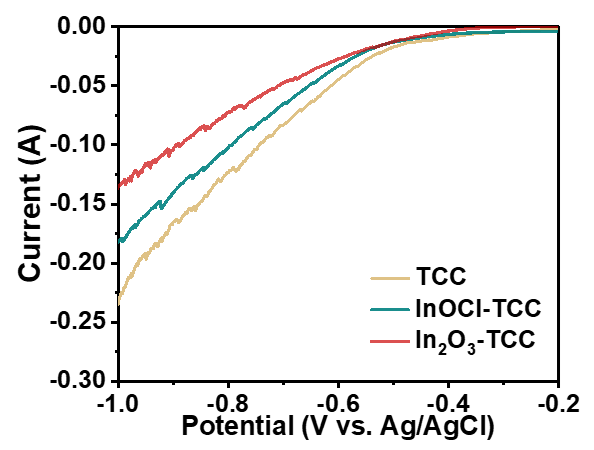


**Fig. S28.** Linear sweep voltammetry (LSV) test of different electrodes.


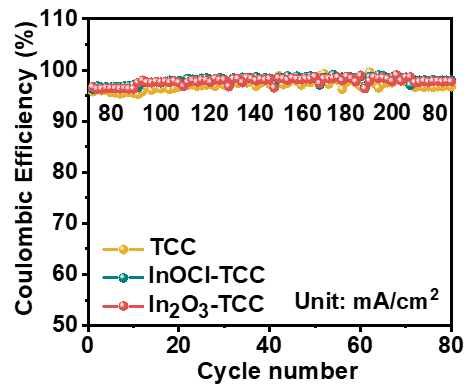


**Fig. S29.** CE of ICRFBs with different electrodes with various current densities.

**Fig. S30.** (a) The Charge-discharge curves, (b) VE, (c) CE, and (d) Discharge capacity of different electrodes at a current density of 140 mA/cm^2^.


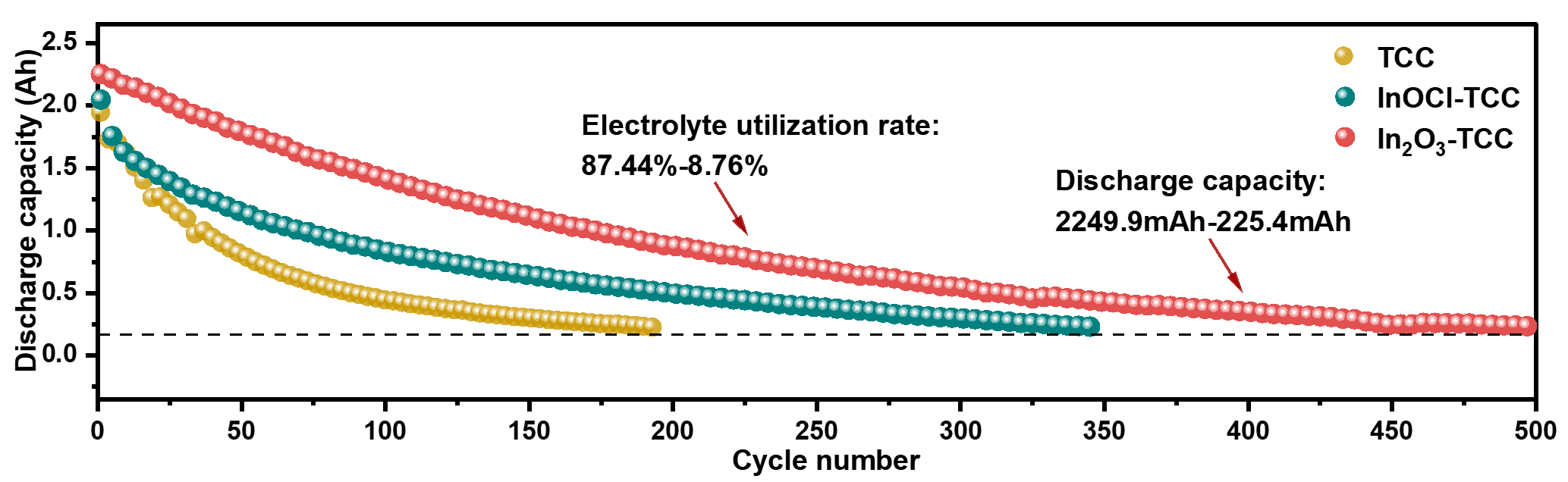


**Fig. S31.** Long cycle discharge capacity and electrolyte utilization of In_2_O_3_-TCC electrode at 140 mA/cm^2^ current density.


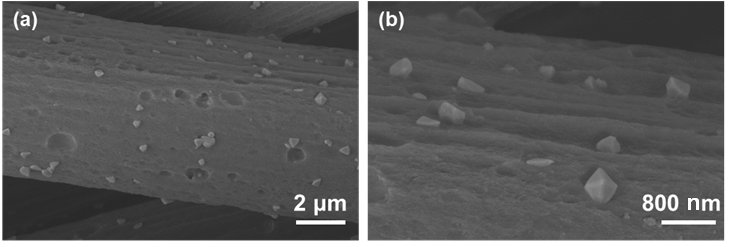


**Fig. S32.** Scanning electron microscopy of In_2_O_3_-TCC electrode after cycling.

**Reference**

[1] Huan Z, Sun C, Ge M. Progress in profitable Fe‐based flow batteries for broad‐scale energy storage[J]. Wiley Interdisciplinary Reviews: Energy and Environment, 2024, 13(6): e541.
